# Optimization of Supercritical Carbon Dioxide Extraction of Polyphenols from Black Rosehip and Their Bioaccessibility Using an In Vitro Digestion/Caco-2 Cell Model

**DOI:** 10.3390/foods12040781

**Published:** 2023-02-11

**Authors:** Kadriye Nur Kasapoğlu, Johanita Kruger, Aslı Barla-Demirkoz, Mine Gültekin-Özgüven, Jan Frank, Beraat Özçelik

**Affiliations:** 1 Department of Food Engineering, Faculty of Chemical and Metallurgical Engineering, Istanbul Technical University, 34469 Istanbul, Turkey; 2 Institute of Nutritional Sciences, University of Hohenheim, Garbenstraße 28, 70599 Stuttgart, Germany; 3 Department of Nutrition and Dietetics, Faculty of Health and Sciences, Halic University, 34060 Istanbul, Turkey

**Keywords:** green extraction, response surface modeling, polyphenols, anthocyanins, bioaccessibility, antioxidant activity

## Abstract

The fruits of *Rosa pimpinellifolia* are rich sources of (poly)phenols, however they are underutilized due to the limited information available. The influence of the pressure, temperature, and co-solvent concentration (aqueous ethanol) of the supercritical carbon dioxide extraction (SCO_2_-aqEtOH) on the extraction yield, total phenolic-, total anthocyanin-, catechin-, cyanidin-3-*O*-glucoside contents, and total antioxidant activity of black rosehip was investigated simultaneously. The maximum obtained total phenolic and total anthocyanin contents under the optimized extraction conditions (280 bar, 60 °C and 25% ethanol, *v*/*v*) were 76.58 ± 4.25 mg gallic acid equivalent and 10.89 ± 1.56 mg cyanidin-3-*O*-glucoside equivalent per g of the dry fruits, respectively. The optimal extract obtained by SCO_2_-aqEtOH was compared to two other extraction procedures: ultrasonication using ethanol as solvent (UA-EtOH) and pressurized hot water extraction (PH-H_2_O). The bioaccessibility and cellular metabolism of the phenolic compounds in the different black rosehip extracts were assessed using an in vitro digestion coupled with a human intestinal Caco-2 cell model. The in vitro digestive stability and cellular uptake of the phenolic compounds had no significant difference among the different extraction methods. The results of this study confirm the efficiency of SCO_2_-aqEtOH extraction for phenolic compounds and, in particular, for anthocyanins, and could be used to produce new functional food ingredients from black rosehip with high antioxidant power containing both hydrophilic and lipophilic compounds.

## 1. Introduction

The use of large amounts of organic solvent in extraction processes is problematic due to health and environmental concerns. In addition, some organic solvents are unsuitable for applications in pharmaceutical and food industries due to their toxicity [1]. Eliminating the major drawbacks of conventional solvent extraction, supercritical fluid extraction (SFE) is a promising alternative with some additional benefits such as better mass transport, higher yield, faster extraction, and selectivity [2]. 

The SFE process is controlled by the operating parameters, such as pressure and temperature, to modify the physical properties of the solvent (density, viscosity and diffusivity) in order to optimize the extraction of the target compounds. For the SFE extraction of potentially bioactive compounds, a temperature range of 40–60 °C and a pressure range of 200–400 bar are often applied [3]. CO_2_ is the most preferred solvent in SFE as it is in gas state at room temperature, enabling its separation from the final extract when the system is decompressed. Additionally, because of its low critical temperature, it can be utilized to extract reactive and thermally sensitive substances and its use decreases post-processing expenses as solvent removal is no longer necessary [4]. When large amounts of CO_2_ are used, the operation can be controlled to recycle it, which is quite advantageous on an industrial scale. Preeminently, CO_2_ has GRAS (Generally Recognized as Safe) status, providing extracts that are safe for human health [5].

When using SFE, the assistance of a modifier co-solvent is needed as the polarity of CO_2_ is low, making it less effective in extracting more polar bioactive compounds. When added in small amounts, methanol or ethanol can induce substantial changes in its solvent properties and can yield the successive extraction of phenolic compounds from plant matrices [6]. Although it is greener than most of organic solvents, large amounts of ethanol-rich solutions are still of concern in both pilot- and industrial-scale productions [7].

The optimization of green and sustainable extraction techniques, such as supercritical fluid technology, is therefore of great value to isolate potentially bioactive compounds from underutilized plants. In that respect, the pseudo fruits of *Rosa pimpinellifolia*, or black rosehips, are promising sources of a variety of potentially bioactive compounds, including flavonoids, anthocyanins, carotenoids, vitamins E and C [8,9,10], and they belong to the third most culturally important family in Turkey: *Rosaceae* [11]. Traditionally, decoction prepared from fruit and root parts of black rosehip are used against colds, infections, stomach pain, hemorrhoids, and also as a cardiotonic agent in the East and North East Anatolian regions [12]. However, black rosehips are less well-known than other *Rosa* species [13]. Several attempts have been made to extend the benefits of locally consumed black rosehips for food industry applications in the forms of vinegar [14], yogurt [15], and ice cream [16]. Sokół-Łętowska et al. reported that black rosehip liqueur had the highest antioxidant activity among liqueurs made from other red fruits, including chokeberry, cornelian cherry, blackcurrant, blackberry, raspberry, mahonia, sloe, strawberry, and sour cherry [17]. In the literature, only a small number of papers deal with the extraction of black rosehip (poly)phenols, using novel techniques, such as microwave assisted aqueous two-phase extraction [18] and ultrasound assisted extraction [19]. Ultrasound and microwaves are recognized as outstanding energy sources that enhance the extraction process, contributing to a good yield and quality of the extract [20]. As a nonconventional technique, pressurized hot water extraction was recently conducted on black rosehip by our group [21]. To the best of our knowledge, supercritical carbon dioxide modified by aqueous ethanol as a co-solvent (SCO_2_-aqEtOH) has not been systematically used for the extraction of black rosehip compounds. 

The purpose of this study was to optimize the SCO_2_ process variables for the extraction of (poly)phenolic compounds from black rosehip, and to compare it with other extraction methods with respect to the antioxidant activity, phenolic, and anthocyanin contents. Any potential bioactivity of these compounds is limited by their bioaccessibility in the human gastrointestinal tract, and then their cellular uptake and metabolism [22]. Thus, the bioaccessibility of the phenolic compounds from different black rosehip extracts were also investigated using simulated gastrointestinal digestion, followed by a human intestinal epithelial cell model. To the best of our knowledge, this is the first study to compare the phenolic content and antioxidant activity of different black rosehip extracts before and after in vitro digestion. In addition, the effects of the extraction method on the cytotoxic properties of extracts were evaluated.

## 2. Materials and Methods

### 2.1. Plant Materials

Black rosehip (*Rosa pimpinellifolia* L., syn. *Rosa spinosissima* L.) fruits were obtained from Gümüşhane Province in Blacksea region of Turkey. The morphology of the fresh fruits are shown in Supplementary Figure S1. The collected fresh fruits were cleaned and the stalk parts were removed. Whole fruits consisting of flesh and seeds were milled using liquid nitrogen and lyophilized overnight to adjust the residual moisture content to below 5% (Christ Alpha 1-2 LD plus, Buch and Holm, Herlev, Denmark). The average particle size of the feed was about 1 mm (corresponding to 18 mesh). The samples were stored at −20 °C until extractions and analyses.

### 2.2. Supercritical Carbon Dioxide Modified by Aqueous Ethanol as Co-Solvent (SCO_2_-aqEtOH)

The supercritical carbon dioxide (CO_2_) extraction process was performed using a supercritical fluid extraction system (Waters SFE 1000, Milford, MA, USA). In order to find the optimal experimental conditions for black rosehip, a response surface design was employed for the simultaneous maximization of all the investigated responses (extraction yield, total phenolic-, total anthocyanin-, catechin-, cyanidin-3-*O*-glucoside contents, and total antioxidant activity). The extraction procedure was carried out dynamically for 60 min at a constant mass flow rate of 50 g min^−1^ of CO_2_ (co-solvent is 10%), in which 12 g of lyophilized samples were used for each run. A Box-Behnken experimental design (BBD), considering three factors and three levels, was selected in order to optimize the following process parameters: pressure (X_1_; 150–350 bar), temperature (X_2_; 40–60 °C), and co-solvent concentration (X_3_; 20–100% EtOH:H_2_O) (Supplementary Table S1). The dependent variables Y_1_ (extraction yield,%, *w*/*w*), Y_2_ (total phenolic content -TPC, mg GAE/g), Y_3_ (catechin content -CC, mg/g), Y_4_ (total anthocyanin content -TAC, mg Cy3GE/g), Y_5_ (Cy3GC content, mg/g), Y_6_ (total antioxidant activity –TAA, mmol TE/g by DPPH assay and Y_7_ (total antioxidant activity –TAA, mmol TE/g by CUPRAC assay were evaluated in the extraction process optimization. The design consisted of 17 experimental runs and was performed in a randomized order (Table 1). The results were statistically analyzed using the Design Expert (Version 13) software to evaluate the suitability of the proposed model and determine the optimum extraction conditions. The treatment of multiple responses and the selection of the optimum conditions were based on the desirability function D. The model fitting was employed by evaluating the coefficient of determination (R^2^) and lack of fit. The significance was determined for all of the independent variables, their interactions and their quadratic effects, considering *p* < 0.05 as significant.

### 2.3. Ultrasound Assisted Solvent Extraction (UA-EtOH)

Ultrasound assisted (UA) extraction was performed in an ultrasonic bath (Sonorex Digitec DT 255 H, Bandelin instruments, Berlin, Germany) using ethanol (99%) as a solvent. The temperature was controlled by ice bags. Two cycles of 30 min extraction were employed, and the supernatants were combined after centrifugation for 10 min at 5000 rpm (Hettich Rotanta 460R, Tuttlingen, Germany). A total of two static extraction cycles were performed and the supernatants were combined.

### 2.4. Pressurized Hot Water Extraction (PH-H_2_O)

Pressurized hot water extraction was performed using optimized conditions, as reported in our previous work [21]. A bench-scale pressurized solvent extraction system (SFE-500, Separex, Champigneulles, France) was used at 75 °C for 1 h, maintaining a pressure of 100 bar with no agitation. 

### 2.5. Extraction Yield 

The collected extracts were put in glass flasks and weighed in analytical balance. The percentage yield (*w*/*w*) was calculated as the mean value of the ratios between the mass of the extract and the mass of the fruit sample used for the extraction, on a dry weight basis.

### 2.6. Spectrophotometric Assays

#### 2.6.1. Total Phenolic Content (TPC)

The total phenolic content was measured by a colorimetric Folin-Ciocalteu assay, as described earlier [23]. A 1500 µL ten-fold diluted Folin-Ciocalteu’s Phenol Reagent was added into 200 µL of the sample, and then were mixed with 1200 µL of sodium carbonate solution (7.5%, *w*/*v*) at room temperature. The absorbance was read after 45 min at 765 nm in a microplate reader (SynergyHT, Biotek, Winooski, VT, USA). Gallic acid was used for quantification and the results were expressed in terms of the gallic acid equivalent per gram fruit in dry weight (mg GAE per g dw). 

#### 2.6.2. Total Anthocyanin Content (TAC)

The total anthocyanin content of the phenolic-rich extracts was estimated using the pH differential method [24]. Aliquots of each sample were diluted with 0.025 M potassium chloride buffer (pH 1) and 0.4 M sodium acetate (pH 4.5), respectively. The absorbance of each dilution was measured at 520 and 700 nm, respectively. The anthocyanin concentrations were expressed as cyanidin-3-*O*-glucoside equivalents (mg Cy3GE per g) = (A × MW × DF × V)/ɛ × W × 0.75, where A = (A520 nm–A700 nm)pH 1.0 − (A520 nm–A700 nm)pH 4.5, MW = 449.2 g/mol for Cy3G, DF = dilution factors, V = extract volume, ε = 26,900 L/mol extinction coefficient, W = plant sample weight, 0.75 = pathlength (cm). 

#### 2.6.3. Total Antioxidant Activity (TAA) 

The hydrogen-donating or radical-scavenging ability of the samples was measured using the stable radical DPPH (2,2-diphenyl-1-picryl-hydrazyl), according to Altin et al. [23], and CUPRAC (cupric ion reducing antioxidant activity) assays were applied, as described by Apak et al. [25]. All of the results were expressed as mmol of Trolox equivalents per gram of the fruit at dry weight (mmol TE per g dw).

### 2.7. Analysis of Phenolic Compounds

Individual phenolics and anthocyanins in the extracts were detected using a high performance liquid chromatography with a PDA detector (SPD M20A, Schimadzu, Kyoto, Japan) according to our previous work [26]. For the chromatographic separation of the phenolic compounds, an ACE C18 column (250 mm × 4.6 mm, 3 μm) with a guard column (4.0 mm × 10 mm, 2 μm) (Advanced Chroma-tography Technologies Ltd., Aberdeen, UK) was used. The gradient of mobile phase A (MQ-water/formic acid, 99.9/0.1 *v*/*v*) and mobile phase B (acetonitrile) was used. 

The chromatographic separation of anthocyanins was performed on a Luna-5μ-Phenyl-Hexyl column (250 mm × 4.6 mm, 5 μm) (Phenomenex, Torrance, CA, USA) using a gradient of MQ-water/formic acid (95:5 *v*/*v*) for mobile phase A and acetonitrile for mobile phase B, as previously described [26]. 

### 2.8. Analysis of Lipophilic Compounds

For the chromatographic analysis of the liposoluble compounds, the extraction and quantification were performed as described previously in detail [27]. For the analysis of carotenoids, ethanolic suspensions were diluted in acetonitrile–methanol–water (85:10:5, *v*/*v*) and injected into the Shimadzu HPLC system, as described in their study. The standards including β-carotene, α-carotene, β-cryptoxanthin, lutein, zeaxanthin and lycopene used for the identification of carotenoids and the UV–visible detector was set to 450 nm for quantification.

For the analysis of vitamin E, the samples suspended in the methanol: ethanol (80:20, *v*/*v*) suspension were injected into a JASCO HPLC system, as previously described [28]. The excitation/emission wavelengths of 296/325 nm were used in the fluorescence detector. The identification of vitamin E congeners was performed by comparing the retention times with those of the authentic standards of α-, β-, δ-, γ-tocopherol and α-, β, δ-, γ-tocotrienol.

### 2.9. In Vitro Gastrointestinal Digestion

The in vitro digestion protocol was performed as described previously in detail [21]. The final digests were centrifuged for 20 min (13,300× *g*, 4 °C) and then filtered through 0.2 μm membrane filters (Filtropur S, Sarstedt, Germany) to separate the soluble and bioaccessible fractions, respectively. All of the digested fractions were overlaid with a layer of nitrogen gas and stored at −80 °C before HPLC analysis. The digestive stability (%) refers to the amount of compound remaining in the whole digest (not degraded during digestion), as a percentage of the total amount of the compound digested. The aqueous solubility (%) is the amount of compound solubilized after digestion (amount in supernatant after centrifuging), as a percentage of the total amount of compound digested. The bioaccessibility (%) is the amount of compound recovered in the digest after centrifuging and filtration, and is expressed as the percentage of the total amount of the compound initially added to the digestion (µg/mL).

### 2.10. Cell Culture

Caco-2 cells, a human colon adenocarcinoma cell line (American Type Culture Collection, Manassas, VA, USA), were used 14–16 days post-confluence. The Caco-2 cells were cultured in DMEM, containing 10% foetal bovine serum, 1% sodium pyruvate, 1% non-essential-amino-acids and 1% penicillin/streptomycin at 5% CO_2_ and 37 °C. All of the experiments were conducted between passages 12 and 46 with replicates from three different passages.

### 2.11. Cytotoxicity

The cytotoxicity of the extracts (to evaluate possible toxic effects of chemical compounds from extracts) and their digests (to determine the appropriate dilutions before uptake studies) were evaluated using a neutral red uptake (NRU) assay in differentiated Caco-2 cells, according to Flory et al. [29]. The results were expressed as the percentage of viable cells, with 100% representing the control cells treated with only PBS.

### 2.12. Caco-2 Uptake of (poly)phenols

The intestinal uptake of phenolics was estimated according to Kruger et al. [30]. The bioaccessible fraction of the in vitro digestion was diluted with DMEM at a ratio of 1:6 (*v*/*v*) and incubated at 37 °C in a humidified atmosphere of 5% CO_2_ for 3 h. After the incubation, the supernatant was removed and stored at −80 °C and the cells were rinsed twice with PBS. The cells were detached using a cell scraper, covered with nitrogen gas, and stored at −80 °C before HPLC analysis. The Caco-2 cells were deproteinized by vortexing with 2 mL ethanol for 30 s and centrifuged (1690× *g* for 5 min, 4 °C). The supernatant was dried under vacuum and the residues were resuspended in 1 mL of PBS. To analyze the free and conjugated catechin and epicatechin, all of the samples were subjected to glucoronidase hydrolysis, extracted using ethyl acetate and analyzed by the JASCO HPLC system, as previously described [30]. The cellular uptake results represent the amount of phenolic absorbed by the Caco-2 cells (µg/well) and is expressed as the percentage of phenolic that remained stable during the incubation period (sum of (poly)phenols in cell lysate and supernatant).

### 2.13. Statistical Analysis

Statistical analysis of the RSM design results was implemented using Design Expert 13.0 (Stat-Ease, Inc., Minneapolis, MN, USA). Unless otherwise stated, the data were obtained from at least three independent experiments and reported as mean ± standard deviation (SD). The differences between the means were determined using one way analysis of variance (ANOVA) with Tukey’s post-hoc test at 95% confidence level using Minitab 16 (Minitab Inc., State College, TX, USA).

## 3. Results and Discussion

### 3.1. Model Fitting and Optimization

The optimization of supercritical carbon dioxide modified by aqueous ethanol as co-solvent extraction conditions were carried out to improve the extraction efficiency of the black rosehip (poly)phenols. A three-level and three factor Box-Behnken design (BBD) was implemented for the response surface optimization of the SCO_2_ extraction process. The operating variables and measured responses in the 17 experiments are given in Table 1. The results of the ANOVA and regression analyses are presented in Supplementary Table S2. In this study, model terms with *p* < 0.01 are highly significant, those with 0.01 ≤ *p* < 0.05 are significant and those with *p* ≥ 0.05 are insignificant [31]. In order to develop the RSM models, the response values were fitted to a second-order polynomial equation using the Design Expert 13.0 software. The nonsignificant factors are excluded from the polynomial equation (*p* > 0.05). The mathematical equations relating the experimental data to the process parameters, expressed in coded values, are presented in Equations (1)–(7):

(1) $$Y=-82.92204+0.007125X_{1}-3.46039X_{2}-0.474967X_{3}-0.032829X_{2}^{2}\;-0.003771X_{3}^{2}+0.000625X_{1}X_{2}-0.000394X_{1}X_{3}\;-0.0025X_{2}X_{3}$$

(2) $$TPC=-236.30025+0.509462X_{1}+6.45763X_{2}+2.29167X_{3}-0.000812X_{1}^{2}\;-0.044710X_{2}^{2}-0.012880X_{3}^{2}-0.001031X_{1}X_{3}\;-0.022744X_{2}X_{3}$$

(3) $$\mathrm{CC}=1.972965+0.002359X_{1}+0.018750X_{2}+0.046558X_{3}\;-0.000019X_{1}^{2}-0.000409X_{3}^{2}+0.000145X_{1}X_{2\;}\;-0.000794X_{2}X_{3}$$

(4) $$\mathrm{TAC}=-28.79900+0.017400X_{1}+1.34125X_{2}+0.143763X_{3}-0.000140X_{1}^{2}\;-0.014660X_{2}^{2}-0.001151X_{3}^{2}+0.001080X_{1}X_{2}\;-0.000102X_{1}X_{3}-0.0016787X_{2}X_{3}$$

(5) $$\mathrm{Cy}3\mathrm{GC}=-1.31336+0.030458X_{1}-0.091875X_{2}-0.162049X_{3}\;-0.0000057X_{1}^{2}-0.001364X_{3}^{2}-0.001606X_{2}X_{3}$$

(6) $$\mathrm{TAA}-\mathrm{DPPH}=-602.23625+0.617125X_{1}+23.05500X_{2}+2.50650X_{3}\;-0.001073X_{1}^{2}-0.182075X_{2}^{2}-0.014942X_{3}^{2}-0.051062X_{2}X_{3}$$

(7) $$\mathrm{TAA}-\mathrm{CUPRAC}=-718.50461+2.35178X_{1}+17.84375X_{2}+18.39224X_{3}\;-0.003741X_{1}^{2}-0.125680X_{3}^{2}-0.005412X_{1}X_{3}\;-0.181000X_{2}X_{3}$$

In the mathematical models developed for each response variable, the coefficients of the terms X_1_, X_2_, and X_3_ illustrate the influence of the independent variables’ operating pressure, temperature, and co-solvent concentration, respectively, and their interactions. The mixtures of ethanol/water were tested as a co-solvent concentration based on the proportion of ethanol in order to determine the effect of using different compositions of the co-solvent. The detailed investigation of the variance and accuracy of the models is summarized and presented in Table S2. The R^2^ values were above 0.95 for all of the investigated responses and the lack of fit was non-significant, implying that all of the models accurately predicted the related responses (*p* > 0.05). Notably, the linear coefficients (X_2_ and X_3_) and quadratic term coefficients (X_2_^2^ and X_3_^2^) greatly influence the overall extraction yield (*p* < 0.05), while the effect of the other term coefficients is moderate (*p* > 0.05). The statistical analyses indicated the extent of the impact of the main variables on the extraction yields following the order: co-solvent concentration < extraction temperature < extraction pressure.

### 3.2. Effects of SCO_2_ Extraction Process Parameters on the Responses

**Figure 1:**
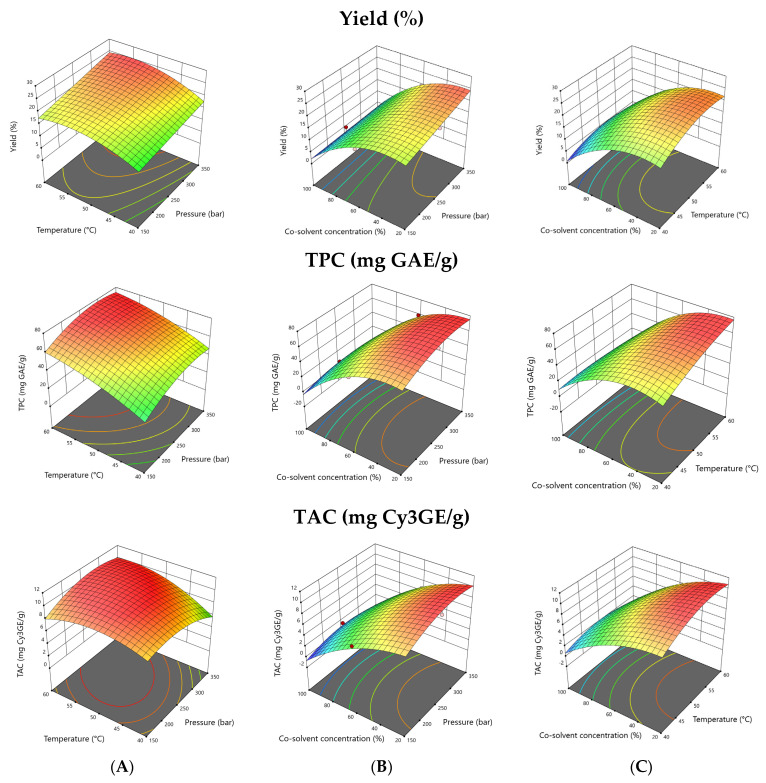
Response surface plots for the interactive effects of (**A**) pressure and temperature; (**B**) extraction pressure and co-solvent concentration; (**C**) temperature and co-solvent concentration on the extraction yield, total phenolics content (TPC) and total anthocyanin content (TAC) recovered from black rosehip. The excluded variable was fixed at its optimal point.

**Figure 2:**
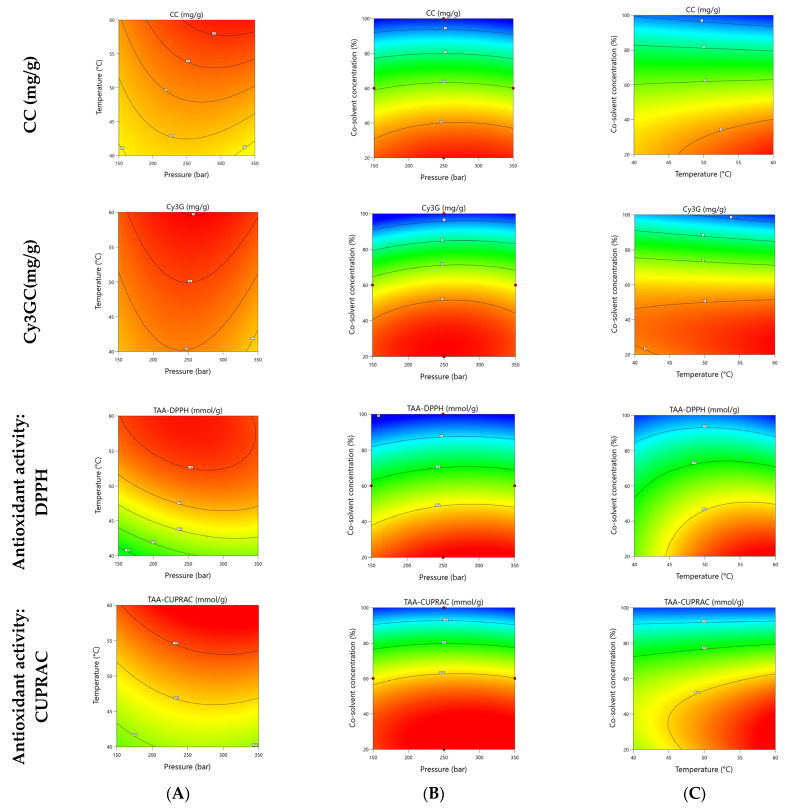
Contour plots for the interactive effects of (**A**) pressure and temperature; (**B**) extraction pressure and co-solvent concentration; (**C**) temperature and co-solvent concentration on the catechin- (CC), cyanidin-3-O-glucoside contents (Cy3GC), and total antioxidant activity (DPPH and CUPRAC assays) in black rosehip. The excluded variable was fixed at its optimal point.

Graphical illustrations of the 3D response surfaces and contour plots showing the regression equations visually are given in Figure 1 and Figure 2. The co-solvent concentration and extraction temperature were the dominant factors for all of the investigated responses. As shown in Figure 1, the extraction yield increases with the increasing pressure and temperature, while decreasing the co-solvent concentration (the proportion of ethanol within co-solvent). The yield of the extraction process was in the range of 2.4–25.1% under the investigated operation conditions (Table 1). The co-solvent concentration was the most effective parameter on the extraction yield in SCO_2_ (*p* < 0.05), which is in agreement with the results reported by Monroy et al. [32]. Being green solvents with the possibility of direct use in edible formulations, water and ethanol are commonly preferred due to their low cost. As co-solvents, water-ethanol mixtures have also been demonstrated to be more efficient for obtaining extracts concentrated in phenolic compounds [33]. The highest extraction of phenolic compounds was obtained at the lowest co-solvent percentages used, when the molar fractions of water and ethanol are the lowest and those of carbon dioxide the highest [34].

When the temperature increased from 40 to 60 °C, the phenolic and anthocyanin content of the extracts was increased (Figure 1). This is because raising the temperature in SCO_2_ extraction using ethanol as a modifier enhances the solubility of (poly)phenols, eventually increasing their extraction. Similar observations were reported in previous studies [35]. As can be seen in Table 1, the phenolic content increased from 50.92 to 63.59 mg GAE/g when the pressure rose from 150 to 250 bar (at 50 °C with co-solvent concentration 20%). However, the total phenolic content and the total anthocyanin content decreased from 4.93 to 1.10 mg GAE/g and from 1.25 to 0.13 mg Cy3GE/g, respectively, when the co-solvent concentration was 100%. The plots showed that the total phenolic and anthocyanin yield increased with the increasing pressure until a specific point, and then decreased if the pressure was raised further. A rise in the pressure can increase the SCO_2_ fluid density, and can thereby strengthen the interactions between the supercritical fluid and the raw material [36]. Table S2 shows a highly significant positive linear effect of the co-solvent concentration (*p* < 0.01) on the recoveries of the total phenolic-, catechin- and cyanidin-3-*O*-glucoside contents.

Figure 2 displays the effects of the SCO_2_ extraction conditions on antioxidant activity, assessed by the DPPH and CUPRAC assays, respectively. The highest antioxidant power was achieved when 250 bar was applied at 60 °C with a co-solvent concentration of 20% (192.7 and 754.8 mmol TE/g) by the DPPH and CUPRAC assays, respectively. Nevertheless, further increment in the pressure did not produce better results, which is in accordance with the other findings related to the phenolic contents. At a high pressure, the condition may be more favored for complex substances, which may not be responsible for the antioxidant behavior related to DPPH radical scavenging or Cupric ion reducing activity assays. These results are similar to those reported by de Souza et al., who reached the highest phenolic content at 25 MPa from *Arctium Lappa* leaves [37]. In another work, the maximum (poly)phenols yield was extracted at 250 bar from guayusa leaves [38]. 

### 3.3. Verification of Optimal SCO_2_ Extraction Conditions 

For the numerical optimization of the extraction process, the statistical software provided combinations of the factor levels that simultaneously maximize the responses on each dependent variable. The RSM model predicted SCO_2_ conditions of 280 bar pressure, 60 °C temperature, and 25% EtOH concentration when a co-solvent ratio of 10% (representing the proportion of co-solvent in CO_2_) applied. The optimal responses with high desirability values are presented in Table 2. To validate the optimal conditions, an extra extraction run was performed, and the actual values were compared with the predicted values (α = 0.05) and were experimentally verified. In a similar work, factors including pressure (150–300 bar), temperature (40–70 °C), co-solvent ratio (5–15%), and time (30–60 min) were assessed for the SCO_2_ extraction of custard apple peel [39]. The extract had optimal phenolic content and antioxidant activity after 54 min when 261 bar was applied at 52 °C with a co-solvent ratio of 12%. In the study of Bimakr et al., SCO_2_ extracts from spearmint leaves obtained at 60 °C, 200 bar, for 60 min, yielded comparable results with conventional solvent extraction (Soxhlet extraction with 70% ethanol) [40].

### 3.4. Comparison of Supercritical CO_2_ Extraction with Other Extraction Methods

With the aim of comparison, another extraction was performed using anhydrous ethanol as a solvent in sonication extraction (UA-EtOH) for the efficient recovery of both lipophilic and hydrophilic constituents. In addition, the optimal SCO_2_-aqEtOH extract was also compared with PH-H_2_O extract, which was obtained in our previous work [21]. The phenolic and anthocyanin contents of these three black rosehip extracts are shown in Table 2. Although UA-EtOH was superior in terms of the phenolic content and antioxidant activity, the SCO_2_-aqEtOH had the highest anthocyanin content (10.89 ± 1.56 mg Cy3GE/g) among the above mentioned extraction methods. Polar solvents are required for the effective extraction of anthocyanins as they occur as glycoside forms [41]. Despite the higher ability of water to extract anthocyanins being expected, a lower efficiency of anthocyanin extraction by PH-H_2_O can be explained by the static mode of extraction in the applied procedure [21]. Odabaş and Koca reported optimal microwave assisted aqueous two-phase extraction conditions to isolate black rosehip anthocyanins. An amount of 13.73 mg Cy3GE/g dry fruit was achieved when 26.85% ethanol (*w*/*w*) with 19.15% ammonium sulfate (*w*/*w*) were used, whereas only 80% ethanol (*v*/*v*) as a solvent yielded 14.85 mg Cy3GE/g fruit. However, the authors noted that the use of the ethanol/ammonium sulfate aqueous two-phase system improved the purity of the extract significantly [18]. 

Taking the obtained values into consideration (Table 2), the performance of the studied dynamic SCO_2_-aqEtOH extraction procedure is better than static PH-H_2_O extraction and comparable with those obtained by UA-EtOH to obtain black rosehip (poly)phenols. The higher extraction efficiency of ethanol together with ultrasonication could be attributed to the phenomenon of cavitation, which occurred in the solvent by the passage of an ultrasonic wave [42]. Zor et al. prepared black rosehip flesh extracts using water and acid–added aqueous ethanol using both conventional and ultrasonication methods. The water extracts revealed higher DPPH free radical scavenging activity, independent from the method of extraction [16]. It has been well-established that the choice of the extracting solvent—in other words, the solvent polarity—has significant effects on the extraction yield of phytochemicals [43,44]. Methanol and ethanol are two efficient choices of co-solvents as they are both soluble in supercritical CO_2_. Although methanol is the solvent with the best results for phenolic compounds, ethanol was preferred, due to safety reasons, for food and environment applications [45]. The use of methanol as a solvent in comparison to ethanol was also investigated by means of UA and maceration extractions. Supplementary Table S3 shows the trend of the yield, TPC, and TAC in black rosehip extracts obtained via different methods and solvents including methanol. The composition of the major phenolic compounds from the black rosehip extracts analyzed by LC-MS/MS are given in Supplementary Figure S2. The individual lipophilic compounds in different black rosehip extracts and fruit parts are summarized in Table 3.

Carotenoids and vitamin E congeners are lipophilic constituents present in rosehip and may differ in content or composition owing to genetic and time variations, the degree of maturation and the analytical protocol [8]. The results in Table 3 suggested that SCO_2_ extraction modified by aqueous ethanol as a co-solvent is capable of dissolving a greater variety of phenolic compounds. β-carotene was the major carotenoid in all of the black rosehip samples, followed by zeaxanthin, with 24.5–7.0 times lower concentrations in the SCO_2_-aqEtOH extract than the UA-EtOH extract (Table 3). On the other hand, the vitamin E content of the SCO_2_-aqEtOH extract was significantly higher than that of the UA-EtOH extract (*p* < 0.05) due to the smaller lipophilic nature of α-tocopherol than β-carotene. The tocopherol content of the black rosehip flesh was 136.93 ± 1.09 µg/g dw. Andersson et al. found a mean annual tocopherol content of 187.6 ± 16.9 μg/g dw in seedless black rosehip sampled at different harvesting times, which is also higher than in other *Rosa* species, such as *R. rubiginosa* and *R. dumalis* [9].

### 3.5. Bioaccessibility and Caco-2 Uptake of Phenolic Compounds

The phenolic compounds that were predominantly present in the black rosehip extracts were measured in the bioaccessibility and cellular uptake studies. The in vitro digestive stability, solubility, and bioaccessibility of the phenolic compounds from the SCO_2_-aqEtOH, UA-EtOH and PH-H_2_O extracts are displayed in Table 4. The in vitro digestive stability of all the phenolic compounds was low (24.33–44.47%), with no significant differences between the different extraction methods (*p* > 0.05), with the exception of vanillin. The digestive stability of vanillin from the UA-EtOH extract was significantly lower. The in vitro solubility of catechin was the highest (≈40%), followed by epicatechin (30.29–34.17%), vanillin (25.45–30.19%) and quercetin-3-*O*-glucoside (20.96–26.72%). The quercetin-3-*O*-glucoside was also the only phenolic that had a difference between the in vitro solubility from the different extracts, and that from the PH-H_2_O extract was significantly lower than that from the SCO_2_-aqEtOH extract. The changes in the phenolic compound profile after in vitro digestion (Supplementary Table S4) may occur due to the interactions with other substances in the gastrointestinal environment [46]. Despite the isolated differences, it is important to note that there were no overall differences (*p* > 0.05) between the digestive stability and in vitro solubility of the different phenolic compounds.

As expected, the bioaccessibility of the phenolics was slightly lower than the solubility, and, overall, there was no difference between the bioaccessibility of the phenolics from the SCO_2_-aqEtOH and UA-EtOH extracts (*p* > 0.05). The in vitro digestion studies are important because they estimate the actual amount of soluble and accessible phenolic compounds available for absorption and potential biological function at the intestinal level [47]. Interestingly, there was constant differences between the bioaccessibility of the phenolics from the SCO_2_-aqEtOH and PH-H_2_O extract. These variations could be accredited to the lower solubility of these phenolic compounds or the affinity of digestive enzymes to these compounds from the PH-H_2_O extract. In contrast to the other extracts, the dried form of the PH-H_2_O extract used in the digestion experiments was obtained via freeze drying, which could lead to the formation of a crystalline structure [48]. The solubility of crystalline substances is lower than amorphous ones as they are thermodynamically stable [49]. The deposition form of the carotenoids has previously been postulated to exert a crucial influence on their bioavailability [50].

The bioaccessibility of quercetin-3-*O*-glucoside was found to be the lowest. Notably, after digestion, a new compound, quercetin, was observed in the extracts, due to the hydrolysis of the glycosidic bonds upon digestion. Hence, the release of the aglycone compound quercetin occurred [51]. The major phenolics present in black rosehip are flavan-3-ols, namely catechin and epicatechin as monomers, as well as oligomers of these two monomers (dimers and trimers) [10]. They are known to be unstable under alkaline intestinal conditions due to chemical changes, including oxidation, polymerization and transformation [52]. Up to 65% loss in the monomeric flavan-3-ols content was found after in vitro digestion (Table 4). A higher loss (around 90%) was reported for pure (+)-catechin [53].

**Figure 3:**
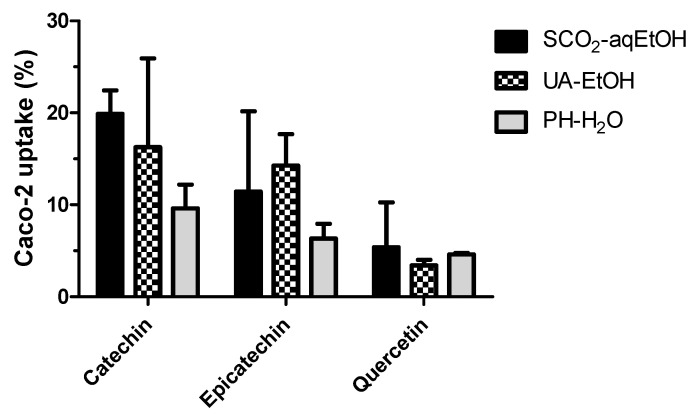
Cellular uptake of phenolics compounds from different black rosehip extracts.

The cellular uptake of the bioaccessible (digested) catechin, epicatechin, and quercetin from the different black rosehip extracts are illustrated in Figure 3. The uptake results of the Caco-2 cell model experiments are calculated as the percentage of the amount taken up in the cells, and the remaining phenolic compounds were stable after the 3 h incubation period. The results indicated that the studied extracts had different degrees of cellular absorption for each phenolic compound. However, no significant differences were observed in terms of the cellular uptake of the phenolic compounds extracted via different methods (*p* > 0.05). A higher degree of cellular absorption correlated with a higher stability of the compounds. In the case of the PH-H_2_O extract, it possessed a considerably lower phenolic absorption rate compared to other two extracts (*p* < 0.05). These phenolic compounds are normally assumed to be absorbed through passive diffusion in Caco-2 cells, where a higher concentration presented to the cells would result in increased cellular uptake [54]. A linear relationship between the dose and uptake was also suggested for chlorogenic acid [55].

### 3.6. Cytotoxicity of Black Rosehip Extracts

The cytotoxicity of the extracts was determined with Caco-2 cells using the neutral uptake assay. The histogram reported in Supplementary Figure S3 represents the viability of the cells after 3 h of incubation with black rosehip extracts obtained via different methods and grape pomace extract obtained using ultrasound assisted ethanol extraction, included in the medium in different amounts and expressed as mg/mL. The extracts substantially reduced the viability of the Caco-2 cells after incubation for 3 h (*p* < 0.05), with no significant differences among extraction methods (*p* > 0.05). Indeed, given 80% viability as the lowest acceptable limit, all of the extracts were shown to be safe up to a concentration of 15 mg/mL. In the literature, the cytotoxic potential of plant extracts is usually studied at lower concentrations and for longer time periods, such as 24 h. In this study, higher concentrations were applied for a shorter time period to more closely simulate the conditions during gastrointestinal digestion. In the hydromethanolic extracts of grape pomace, a concentration higher than 400 µg/mL was reported to achieve a 50% growth inhibition in human tumor cell lines in 24 h [56]. Salau, Yakubu, and Oladiji investigated the cytotoxic activity of the aqueous extracts of two traditional herbs: *Anogeissus leiocarpus* and *Terminalia avicennioides* root barks in Ehrlich Ascites Carcinoma cells for 3 h and 24 h. They reported 80% viability for approximately 10 and 5 µg/mL of plant extract in 3 h and 24 h incubation, respectively [57].

## 4. Conclusions

Our results highlight the importance of co-solvent polarity and temperature as the main experimental factors determining the yield of (poly)phenolic compounds when using SCO_2_ extraction. The optimal extract contained a superior content of anthocyanin and comparable amounts of phenolic content to those obtained by UA-EtOH, without the need to use vast amounts of organic solvents. The nature of the applied procedure and the solvent, overall, determined the selectivity of the extraction process. Hence, aqueous ethanol modified supercritical carbon dioxide and ultrasound assisted extraction were the best ‘green’ methods in our experimental settings for the recovery of phenolic compounds from black rosehip. However, the use of anhydrous ethanol as a solvent is not recommended for anthocyanin extraction. Furthermore, the bioaccessibility and cellular uptake of the individual phenolic compounds did not differ significantly among the tested extracts. The optimized models provide important knowledge about the supercritical extraction of black rosehip using a carbon dioxide + ethanol/water mixture as a solvent for future studies in terms of a process scale-up. However, more research is needed in this area to obtain a more complete profile of the phenolic composition of the obtained extracts. Future studies should focus on the investigation of other ’green’ solvents to further improve the extraction efficiency for hydrophilic and lipophilic bioactive compounds from black rosehip. Being rich in a variety of (poly)phenolic compounds, black rosehip extracts can be used as an alternative source to provide natural additives in potential food and beverage formulations, as well as dietary supplements.

## Figures and Tables

**Table 1:** Experimental conditions and responses obtained defined by the BBD design matrix for the extraction yield, phenolic-, anthocyanin content, and antioxidant activity in black rosehip.

	Factors			Experimental Values						
Run	X_1_ (bar)	X_2_ (°C)	X_3_ (%)	Y	TPC	CC	TAC	Cy3GC	TAA^D^	TAA^C^
1	250	50	60	21.6	67.74	3.36	9.04	7.91	136.2	573.9
1	250	50	60	21.6	67.74	3.36	9.04	7.91	136.2	573.9
2	250	50	60	17.8	56.73	3.12	9.15	6.77	122.9	558.2
3	250	40	100	2.4	12.89	0.97	2.03	2.45	15.8	84.0
4	250	40	20	13.7	44.29	3.53	7.98	7.41	112.0	491.4
5	350	50	100	3.1	1.10	0.44	0.13	1.26	8.6	20.3
6	350	40	60	15	45.82	2.83	3.35	7.31	107.1	434.0
7	250	50	60	21.8	55.69	3.06	7.92	6.70	113.3	593.5
8	350	60	60	19.5	58.97	2.99	6.65	7.00	121.1	629.0
9	150	60	60	14.9	48.07	2.74	6.01	5.90	104.4	553.1
10	250	60	20	19.8	72.87	4.59	9.95	9.03	192.7	754.8
11	250	50	60	17.4	54.05	2.95	9.16	7.98	118.7	554.5
12	150	50	100	3.7	4.93	0.46	1.25	1.01	16.7	42.7
13	250	50	60	18.2	60.10	2.86	7.84	7.26	138.6	519.8
14	150	40	60	12.9	32.23	3.16	7.03	6.62	55.4	426.6
15	150	50	20	19.4	50.92	3.83	9.82	8.31	167.9	582.0
16	350	50	20	25.1	63.59	4.17	10.34	7.71	172.0	646.2
17	250	60	100	4.5	5.08	0.76	1.30	1.50	14.8	57.8

Abbreviations: Y, yield% (*w*/*w*). TPC, total phenolic content (mg GAE/g). CC, catechin content (mg/g). TAC, total anthocyanin content (Cy3GE/g). Cy3GC, cyanidin-3-*O*-glucoside content (mg/g). TAA^D^-DPPH and TAA^C^-CUPRAC, total antioxidant activity by DPPH and CUPRAC assays (mmol TE/g). All in dry basis.

**Table 2:** Predicted and experimental values of the response variables at optimal conditions for SCO_2_ and other extraction methods.

Characteristics	Extraction Methods			
	SCO_2_-aqEtOH		UA-EtOH ^2^	PH-H_2_O ^3^
	Predicted	Experimental ^1^	Experimental	
Y (%, *w*/*w*)	22.024	24.05 ± 1.54 ^B^	23.81 ± 0.92 ^B^	27.90 ± 1.60 ^A^
TPC (mg GAE/g)	77.109	76.58 ± 4.25 ^AB^	81.02 ± 3.07 ^A^	68.90 ± 3.94 ^B^
CC (mg/g)	4.453	4.65 ± 0.39 ^A^	4.36 ± 0.86 ^A^	3.90 ± 0.21 ^A^
TAC (Cy3GE/g)	10.598	10.89 ± 1.56 ^A^	6.05 ± 0.75 ^B^	5.28 ± 0.41 ^B^
Cy3GC (mg/g)	9.023	9.05 ± 1.02 ^A^	4.39 ± 0.42 ^B^	4.08 ± 0.51 ^B^
TAA_DPPH_ (mmol TE/g)	190.976	193.2 ± 7.4 ^B^	206.1 ± 11.3 ^AB^	228.3 ± 16.3 ^A^
TAA_CUPRAC_ (mmol TE/g)	789.168	798.2 ± 12.8 ^B^	840.3 ± 17.5 ^B^	930.5 ± 24.4 ^A^

Different letters indicate significant differences between the extracts (*p* < 0.05). ^1^ Extract obtained at optimum conditions of SCO_2_ extraction, 90:10 (CO_2_: aqueous EtOH), solvent-solid ratio 1:25, 60 min (dynamic). Desirability = 0.995; ^2^ Extract obtained by ultrasound assisted ethanol at 24 °C, solvent-solid ratio 1:10, 60 min (static); ^3^ Extract obtained by pressurized hot water at 75 °C, 100 bar, solvent-solid ratio 1:10, 60 min (static); Abbreviations: Y, yield; TPC, total phenolic content; CC, catechin content; TAC, total anthocyanin content; Cy3GC, cyanidin-3-*O*-glucoside content; TAA-DPPH and TAA-CUPRAC, total antioxidant activity by DPPH and CUPRAC assays. All in dry basis.

**Table 3:** Lipophilic compounds (µg/g dw) of black rosehip and its extracts obtained using different extraction methods.

	UA-EtOH Extract	SCO_2_-aqEtOH Extract	Fruit *
	Carotenoids		
Lutein	1.04 ± 0.03	0.18 ± 0.01	1.08 ± 0.16
Zeaxanthin	5.46 ± 0.36	0.77 ± 0.03	4.44 ± 0.67
β-cryptroxanthin	1.28 ± 0.03	0.23 ± 0.01	0.80 ± 0.11
β-carotene	106.90 ± 2.67	4.36 ± 0.18	10.75 ± 0.91
Total carotenoids	114.69 ± 3.09	17.07 ± 1.84	5.53 ± 0.22
	Vitamin E congeners		
δ-Tocotrienol	1.72 ± 0.12	2.87 ± 0.12	nd
β-Tocotrienol	nd	nd	nd
γ-Tocotrienol	0.10 ± 0.05	0.38 ± 0.02	0.48 ± 0.25
α-Tocotrienol	45.53 ± 3.56	121.76 ± 4.92	2.05 ± 1.26
δ-Tocopherol	0.12 ± 0.03	1.07 ± 0.04	0.26 ± 0.04
β-Tocopherol	1.38 ± 0.19	8.57 ± 0.35	0.23 ± 0.07
γ-Tocopherol	2.17 ± 0.23	9.64 ± 0.39	2.99 ± 0.33
α-Tocopherol	5.27 ± 0.27	73.05 ± 2.95	9.49 ± 2.35
Total tocotrienols	47.4 ± 3.6	125.0 ± 5.1	2.5 ± 1.5
Total tocopherols	8.9 ± 0.3	92.3 ± 3.7	13.0 ± 2.3
Total vitamin E	56.4 ± 3.4	217.3 ± 8.8	15.5 ± 3.7

* denotes black rosehip fruit consisting of flesh and seed. nd: not detected.

**Table 4:** In vitro stability, solubility, and bioaccessibility of phenolic compounds from different black rosehip extracts.

Extract	Nondigested (µg/mL)	Stability ^1^ (%)	Solubility ^2^ (%)	Bioaccessibility ^3^ (%)
	Catechin			
SCO_2_-aqEtOH	1070.4 ± 23.9 ^B^	42.94 ± 4.60 ^A^	37.48 ± 7.72 ^A^	30.17 ± 3.12 ^A^
UA-EtOH	1642.5 ± 262.2 ^A^	44.16 ± 4.90 ^A^	39.24 ± 6.01 ^A^	30.32 ± 1.39 ^A^
PH-H_2_O	961.1 ± 37.4 ^B^	43.50 ± 7.51 ^A^	38.15 ± 9.72 ^A^	20.89 ± 4.37 ^B^
	Epicatechin			
SCO_2_-aqEtOH	406.6 ± 52.5 ^A^	39.27 ± 7.15 ^A^	31.28 ± 9.54 ^A^	29.85 ± 5.09 ^A^
UA-EtOH	388.3 ± 98.1 ^A^	36.53 ± 1.90 ^A^	30.29 ± 4.44 ^A^	25.81 ± 1.79 ^AB^
PH-H_2_O	255.0 ± 31.1 ^B^	44.47 ± 7.81 ^A^	34.17 ± 8.42 ^A^	24.35 ± 2.31 ^B^
	Quercetin-3-*O*-glucoside			
SCO_2_-aqEtOH	510.7 ± 10.7 ^A^	28.75 ± 1.52 ^A^	26.72 ± 0.83 ^A^	24.99 ± 1.65 ^A^
UA-EtOH	479.3 ± 4.3 ^B^	28.60 ± 3.23 ^A^	25.82 ± 2.99 ^AB^	20.46 ± 2.09 ^AB^
PH-H_2_O	252.9 ± 12.1 ^C^	24.33 ± 2.34 ^A^	20.96 ± 2.37 ^B^	18.38 ± 2.56 ^B^
	Vanillin			
SCO_2_-aqEtOH	37.6 ± 2.0 ^A^	39.44 ± 1.11 ^A^	26.03 ± 3.70 ^A^	23.82 ± 1.06 ^AB^
UA-EtOH	28.3 ± 3.1 ^B^	28.74 ± 2.29 ^B^	25.45 ± 3.11 ^A^	22.73 ± 1.14 ^B^
PH-H_2_O	21.8 ± 0.4 ^C^	36.14 ± 1.63 ^A^	30.19 ± 0.95 ^A^	27.57 ± 2.63 ^A^

Data are given as mean ± SD (*n* = 6); One Way Analysis of Variance (ANOVA) coupled with the Tukey’s post-hoc analysis to identify means with significant differences (*p* < 0.05) in each extract indicated by different capital letters (same column). ^1^ Defined as the stability ratio of phenolics after in vitro gastrointestinal digestion. ^2^ Defined as the percentage of the soluble phenolics to the initial total phenolic content. ^3^ Defined as the percentage of the soluble phenolics included in small (<200 nm) micelles or particles.

## Data Availability

The data are contained within the article or the Supplementary Materials.

## Supplementary Materials

The following supporting information can be downloaded at: https://www.mdpi.com/article/10.3390/foods12040781/s1, Figure S1: Morphology of fresh black rosehips; Figure S2: Composition of the major phenolic compounds from black rosehip extracts analyzed by LC-MS/MS. MeOH represents the black rosehip extract obtained by maceration using methanol as a solvent for 60 min; Figure S3: Cell viabilities of the Caco-2 cells after the treatment of different black rosehip extracts at varying doses (mg/mL). The bars represent the standard deviation (*n* = 3); Table S1: Independent variables and their coded and actual values used for optimization in Box-Behnken design; Table S2: Analysis of variance (ANOVA) of the regression model for the analyzed responses in SCO_2_-aqEtOH extraction; Table S3: Effect of the extraction method and solvent on the yield, total phenolic content (TPC), and total anthocyanin content (TAC); Table S4: Changes in the phenolic compounds in different black rosehip extracts during in vitro digestion. The results are expressed as µg/mL digesta.
