# Supplementary material for: Optimization of Supercritical Carbon Dioxide Extraction of Polyphenols from Black Rosehip and Their Bioaccessibility Using an In Vitro Digestion/Caco-2 Cell Model
Source: Foods. 2023 Feb 11;12(4):781. doi: 10.3390/foods12040781 (PMC9957028; doi:10.3390/foods12040781)
Supplement: Supplementary file 1 [file foods-12-00781-s001.zip › foods-2178594-supplementary.pdf]

Supplementary Materials

Table S1: Independent variables and their coded & actual values used for optimization in Box-Behnken design.

Independent variable	Symbol	Coded levels		
		−1	0	1
Pressure, bar	X1	150	250	350
Temperature, °C	X2	40	50	60
Ethanol concentration, % EtOH (v/v)	X3	20	60	100

Table S2: Analysis of variance (ANOVA) of the regression model for the analyzed responses in SCO_2-aqEtOH extraction.

Source	Sum of squares							Mean of squares						
	Y1	Y2	Y3	Y4	Y5	Y6	Y7	Y1	Y2	Y3	Y4	Y5	Y6	Y7
Model	785.83	8540.10	25.30	184.795	110.16	52783.73	888400	98.23	1067.51	3.61	20.5328	18.36	7540.53	126900
X1 (bar)	17.40	138.86	0.0072	1.657	0.2592	518.42	1956.25	17.40	138.86	0.0072	0.2340	0.2592	518.42	1956.25
X2 (°C)	27.01	309.51	0.0435	1.557	0.0162	2542.41	39018.21	27.01	309.51	0.0435	6.9320	0.0162	2545.41	390018.21
X3 (%)	516.81	5390.85	22.75	139.246	86.07	43320.96	643890	516.82	5390.85	22.75	3.2821	86.07	43320.96	643900
X1·X2	1.56		0.0841	4.671				1.56		0.0841	4.6711			
X1.X3	9.92	68.06		0.681			1874.89	9.92	68.06		0.6814			1874.89
X2.X3	4.00	331.06	0.4032	1.813	1.65	1668.72	20967.04	4.00	331.06	0.4032	1.8132	1.65	1668.72	20967.04
$X1^2$		277.52	0.1464	10.557	1.39	485.00	5910.03		277.52	0.1464	8.1937	1.39	485.00	5910.03
$X2^2$	45.50	84.17		10.334		1395.84		45.50	84.17		9.0683		1395.84	
$X3^2$	153.67	1788.25	1.80	14.277	20.12	2406.60	170700	153.672	1788.25	1.80	14.2771	20.12	2406.60	170700
Residual	35.86	169.77	0.4246	4.911	2.84	1725.54	6367.16	4.48	21.22	0.0472	0.7015	0.2838	191.73	707.46
Lack-of-Fit	17.27	51.66	0.2794	3.066	1.36	1238.57	3402.17	4.32	12.91	0.0559	1.0219	0.2273	247.71	680.43
Pure Error	18.59	118.11	0.1452	1.845	1.47	486.97	2964.99	4.65	29.53	0.0363	0.4612	0.3685	121.74	741.25
Total	821.68	8709.87	25.72	189.705	113.00	54509.28	894800							

	Y1	Y2	Y3	Y4	Y5	Y6	Y7
R^2-Sq	0.9564	0.9805	0.9815	0.9741	0.9749	0.9683	0.9929
R^2-Sq (pred)	0.7600	0.9133	0.9103	0.7264	0.9267	0.8687	0.9732
R^2-Sq (adj)	0.9127	0.9610	0.9671	0.9408	0.9598	0.9437	0.9873

Table S2: *Cont.*

Source	*F*-value							*p*-value						
	Y1	Y2	Y3	Y4	Y5	Y6	Y7	Y1	Y2	Y3	Y4	Y5	Y6	Y7
Model	21.92	50.30	76.60	29.27	64.70	39.33	179.40	0.0001*	<0.0001*	0.0001*	<0.0001*	<0.0001*	<0.0001*	<0.0001*
X1 (bar)	3.88	6.54	0.1526	2.36	0.9134	2.77	11.83	0.0843	0.0338**	0.7051	0.1683	0.3617	0.1345	0.1307
X2 (°C)	6.03	14.58	0.9222	2.21	0.0571	55.15	65.46	0.0396**	0.0051*	0.3620	0.1809	0.8160	0.0054*	<0.0001*
X3 (%)	115.30	254.03	482.13	198.53	303.30	910.13	75.96	<0.0001*	<0.0001*	0.0001*	<0.0001*	<0.0001*	<0.0001*	<0.0001*
X1·X2	0.3486		1.78	6.65				0.5712	0.1111	0.2146	0.0365**			
X1.X3	2.21	3.21		0.9585			2.64	0.1751	0.0042*		0.3602			
X2.X3	0.8924	15.60	8.55	2.60	5.82	2.65	29.63	0.3725		0.0169**	0.1510	0.0365**	0.0162**	0.1380
$X1^2$		13.08	3.10	11.70	4.89	29.64	8.35		0.0068*	0.1120	0.0111**	0.0515	0.1462	0.0004*
$X2^2$	10.15	3.97		12.90		8.35		0.00129	0.0816		0.0088*		0.0245**	
$X3^2$	34.29	84.27	38.24	20.34	70.92	241.33	241.36	0.0004	<0.0001*	0.0002*	0.0028*	<0.0001*	0.0063*	0.0179**
Lack-of-Fit	0.9286	0.4374	1.54	2.21	0.6167	0.9180	0.92	0.5277	0.7786	0.3484	0.2293	0.7161	0.2555	0.5483

Independent variables: X1, pressure (bar); X2, temperature (°C); X3, co-solvent concentration (%). Dependent variables: Y1, extraction yield (%); Y2, total phenolic content (mg GAE/g); Y3, catechin content (mg/g); Y4, total anthocyanin content (mg Cy3GE/g); Y5, Cy3G (mg/g); Y6, total antioxidant capacity by DPPH assay (mmol TE/g); and Y7, total antioxidant capacity by CUPRAC assay (mmol TE/g) all in dry basis. DF: Degree of freedom; F-Value: Fisher distribution value; *p*-Value: * significance at $p < 0.01$;
** significance at $p < 0.05$

Table S3: Effect of extraction method and solvent on yield, total phenolic content (TPC), and total anthocyanin content (TAC).

Extraction Method	Solvent	Solvent/ solid ratio (mL/g)	Pressure (bar)	Time (min)	Temperature (°C)	Yield (%)	TPC (mg GAE/g)	TAC (mg Cy3GE/g)
PH-H_2O	Water	10	100	60	75	27.90 ± 1.60[ab]	68.90 ± 3.94[d]	5.28 ± 0.40[d]
SCO_2-aqEtOH *	90 CO_2:10 aqEtOH (25%)	25	280	60	60	25.05 ± 1.54[b]	76.58 ± 4.25[c]	10.89 ± 0.56[b]
UA- EtOH	Ethanol	25	-	60	24	23.75 ± 0.82[c]	81.02 ± 2.10[ab]	6.05 ± 0.23[d]
UA- MeOH	Methanol	25	-	60	24	30.95 ± 1.35[a]	89.55 ± 1.60[a]	13.32 ± 0.11[a]
Maceration- EtOH	Ethanol	25	-	60	24	18.50 ± 1.27[c]	71.28 ± 1.42[cd]	5.32 ± 0.12[d]
Maceration- MeOH	Methanol	25	-	60	24	26.56 ± 1.50[b]	78.43 ± 2.04[bc]	9.41 ± 0.17[c]

*denotes dynamic extraction process

Table S4: Changes of phenolic compounds in different black rosehip extracts during *in vitro* digestion. Results are expressed as µg/ mL digesta.

Extract	Nondigested	Digested fraction[1]	Aqueous fraction[2]	Micellar fraction[3]
		Catechin		
SCO_2-aqEtOH	1070.4 ± 23.9^{Ba}	459.9 ± 54.0^{Bb}	401.9 ± 88.1^{Bbc}	323.2 ± 36.5^{Bc}
UA-EtOH	1642.5 ± 262.2^{Aa}	721.5 ± 109.2^{Ab}	638.9 ± 114.1^{Ab}	495.4 ± 60.5^{Ab}
PH-H_2O	961.1 ± 37.4^{Ba}	418.5 ± 77.6^{Bb}	365.9 ± 90.2^{Bb}	238.2 ± 22.3^{Cc}
		Epicatechin		
SCO_2-aqEtOH	406.6 ± 52.5^{Aa}	157.4 ± 19.5^{Ab}	125.4 ± 35.1^{Ab}	119.7 ± 12.8^{Ab}
UA-EtOH	388.3 ± 98.1^{Aa}	140.8 ± 30.6^{ABb}	116.5 ± 30.6^{Ab}	101.5 ± 32.4 ^{Ab}
PH-H_2O	255.0 ± 31.1^{Ba}	114.2 ± 30.2^{Bb}	87.5 ± 24.6^{Abc}	61.8 ± 6.4^{Bc}
		Quercetin-3-O-glucoside		
SCO_2-aqEtOH	510.7 ± 10.7^{Aa}	146.9 ± 10.0^{Ab}	136.4 ± 4.0^{Ab}	127.7 ± 9.7^{Ab}
UA-EtOH	479.3 ± 4.3^{Ba}	137.1 ± 16.3^{Bb}	123.8 ± 14.9^{Abc}	98.1 ± 11.0^{Bc}
PH-H_2O	252.9 ± 12.1^{Ca}	61.5 ± 5.3^{Cb}	52.8 ± 3.6^{Bb}	46.3 ± 4.4^{Cb}
		Vanillin		
SCO_2-aqEtOH	37.6 ± 2.0^{Aa}	14.8 ± 1.4^{Ab}	9.8 ± 1.3^{Ac}	9.0 ± 0.5^{Ac}
UA-EtOH	28.3 ± 3.1^{Ba}	8.4 ± 1.7^{ABb}	7.2 ± 1.4^{Bb}	6.4 ±0.5^{Bb}
PH-H_2O	21.8 ± 0.4^{Ca}	7.9 ± 1.3^{Bb}	6.6 ± 0.1^{Bc}	6.0 ± 0.6^{Bc}

Data are given as mean ± SD (n=6); One Way Analysis of Variance (ANOVA) coupled with the Tukey's post-hoc analysis to identify means with significant differences ($p < 0.05$) in each extracts indicated by different capital letters (same column) and among digestion phase by different lowercase letters. [1] The phenolic amount in the whole digests. [2] Supernatant taken after the digesta was centrifuged (4,700 ×g, 60 min, 4 °C). [3] Supernatant taken after centrifuged digesta filtered (0.2µm).

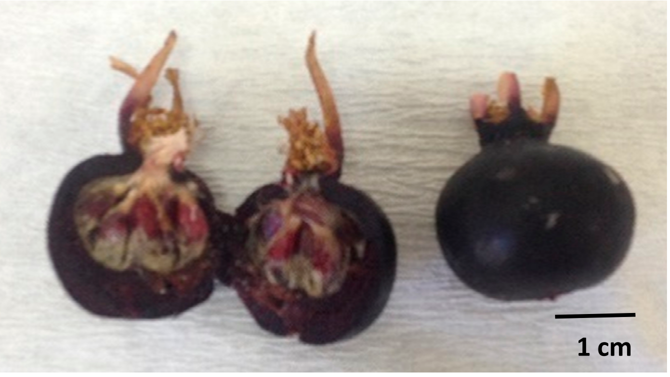

Figure S1: Morphology of fresh black rosehips.

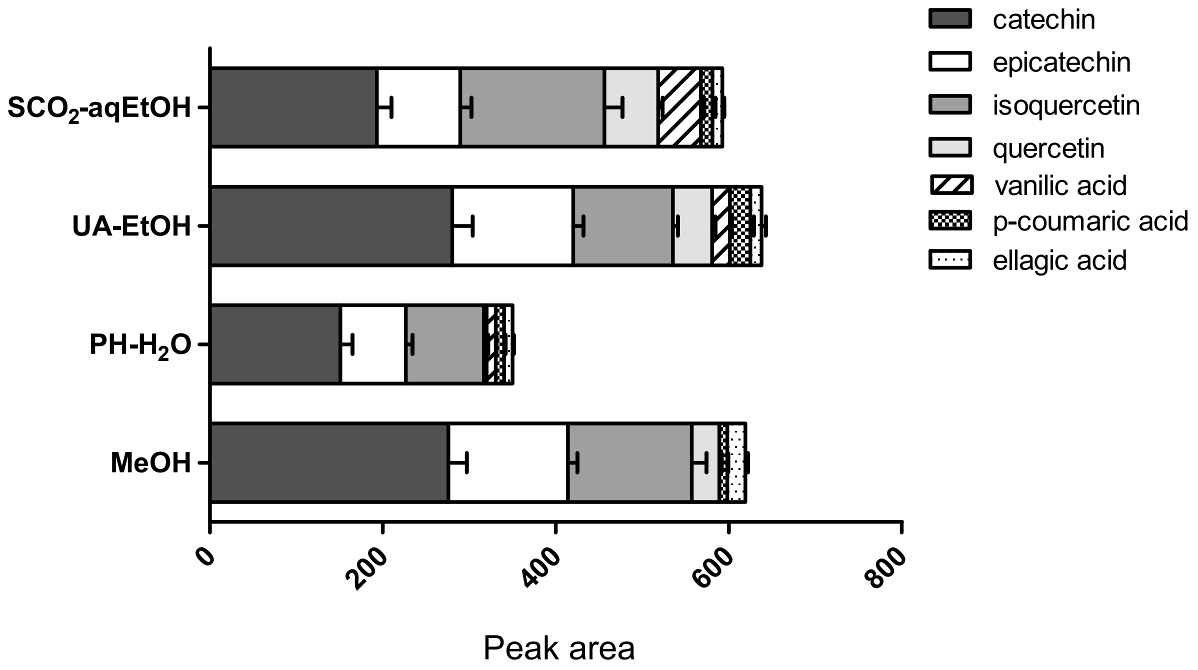

Figure S2. Composition of major phenolic compounds from black rosehip extracts analyzed by LC-MS/MS. MeOH represents black rosehip extract obtained by maceration using methanol as solvent for 60 mins.

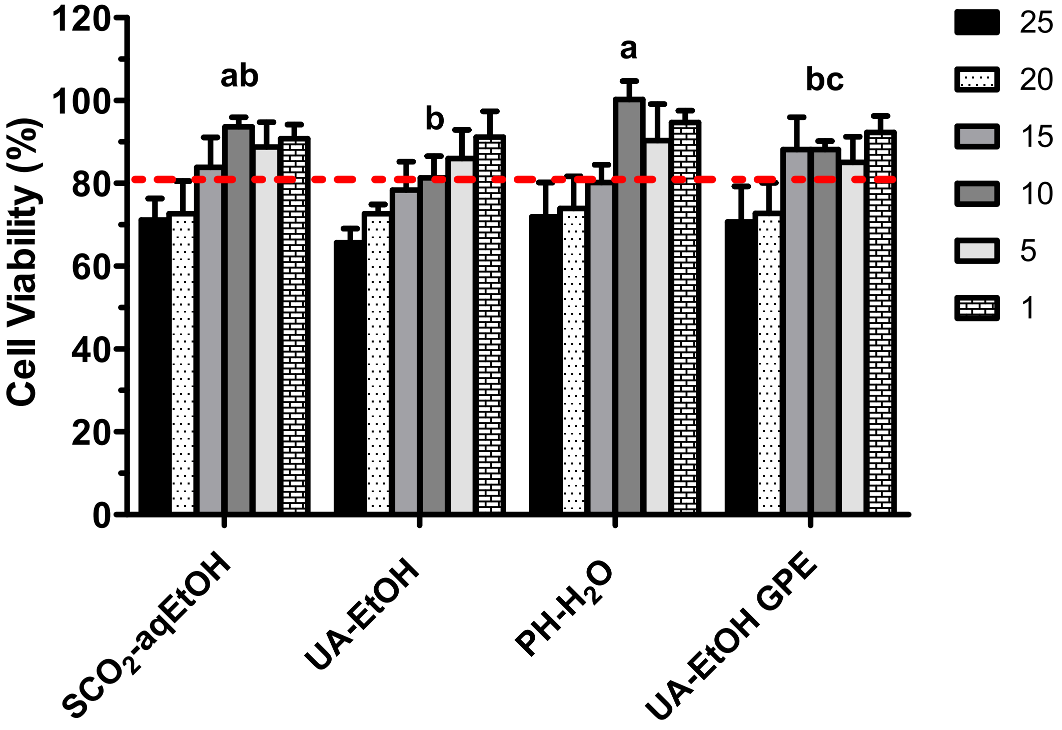

Figure S3. Cell viabilities of Caco-2 cells after treatment of different black rosehip extracts at varying doses (mg/mL). Bars represent standard deviation (n=3).
